# A Multisite Demonstration of Shared Access to Older Adults’ Patient Portals

**DOI:** 10.1001/jamanetworkopen.2024.61803

**Published:** 2025-02-25

**Authors:** Kelly T. Gleason, Catherine M. DesRoches, Mingche M. J. Wu, Danielle Peereboom, Vadim Dukhanin, Timothy W. Farrell, Matthew J. Gonzales, Saloni Sharma, Supriya G. Mohile, Sara Epstein, Mark A. Supiano, Marianne C. Parshley, David L. Roth, Jennifer L. Wolff

**Affiliations:** 1 Johns Hopkins University School of Nursing, Baltimore, Maryland; 2 OpenNotes, Beth Israel Deaconess Medical Center, Boston, Massachusetts; 3 Department of Medicine, Harvard Medical School, Boston, Massachusetts; 4 Department of Health Policy and Management, Johns Hopkins Bloomberg School of Public Health, Baltimore, Maryland; 5 Division of Geriatrics, Spencer Fox Eccles School of Medicine, and the Center on Aging, University of Utah, Salt Lake City; 6 VA Salt Lake City Geriatric Research, Education, and Clinical Center, Salt Lake City, Utah; 7 Institute for Human Caring, Providence, Renton, Washington; 8 Wilmot Cancer Institute, University of Rochester Medical Center, Rochester, New York; 9 Providence Medical Group, Portland, Oregon

## Abstract

**Importance:**

The patient portal has a growing role in health care. Many health systems have implemented shared access functionality in which patients may register a care partner with unique identity credentials to access their portal. Uptake of shared access has been limited.

**Objective:**

To examine the outcomes of a multisite demonstration involving organizational strategies associated with registration and use of patient portal shared access.

**Design, Setting, and Participants:**

This quality improvement study was conducted in diverse sites within 3 health systems in the US: (1) geriatric oncology, (2) geriatric medicine, and (3) primary care. Patients aged 65 years or older with 1 or more visits during the 12-month demonstration period (July 1, 2022, through July 1, 2023, for sites 1 and 2; site 3 was delayed 3 months) and care partners who used the portal accounts of patients meeting these criteria were included. The 6-month postdemonstration period (August 1, 2023, through January 1, 2024) was compared with the 6-month predemonstration period (January 1 through June 1, 2022).

**Exposures:**

Organizational strategies (brochures, webpages, tip sheets, and implementation toolkits created using a human-centered design) to encourage shared access registration and use.

**Main Outcomes and Measures:**

Portal registration and use (logins, laboratory results viewed, clinical notes viewed, visits scheduled) by type of access (patient and care partner) from electronic health record data were examined. Patient- and care partner–reported awareness and use of shared access were measured using a postdemonstration survey.

**Results:**

A total of 16 005 patients from the 3 sites met the inclusion criteria (84.8% younger than 85 years and 61.5% women). Most patients had an activated portal account (91.0%) and logged in at least once (84.8%) during the 24 months spanning the demonstration and pre- and postdemonstration periods. New portal registrations were stable, but nonsignificant for shared access (110 of 14 758 [0.7%] vs 91 of 14 016 [0.6%]) and significantly decreased for patient access (677 of 3158 [21.5%] vs 225 of 1520 [13.2%]). Use of shared access before vs after the demonstration increased for number of logins (mean [SD], 5.9 [11.4] vs 6.8 [14.1]), laboratory results viewed (mean [SD], 0.7 [2.7] vs 1.1 [3.7]), clinical notes viewed (mean [SD], 0.2 [1.1] vs 0.6 [3.2]), and visits scheduled (mean [SD], 0.8 [10.8] vs 1.0 [5.4]). Of the 91 care partners reporting portal use in the postdemonstration survey, 48 (52.7%) indicated primarily using patient credentials and 31 (34.1%) indicated primarily using their own credentials. Less than one-half of patients (721 of 1664 [43.3%]) stated being aware of shared access.

**Conclusions and Relevance:**

These findings show no association of the multisite demonstration with increased new registrations for shared access and only modest increases in portal use among care partners of older adults. As portal use expands to encompass legal documentation, medical decision-making, and patient education, policies to support proper use of identity credentials are needed.

## Key Points

**Question:**

What are the outcomes associated with an organizational initiative to increase registration and use of shared access to the patient portal among care partners of older adult patients?

**Findings:**

In this quality improvement study 16 005 patients from 3 diverse US sites, new shared access registration was unchanged; however, use of shared access functionality among registered care partners increased. Care partners logged in more frequently, viewed more laboratory results and clinical notes, and scheduled more visits after the demonstration.

**Meaning:**

These findings underscore the need for policies and strategies that support proper use of identity credentials as patient portals are increasingly used in managing legal and medical information.

## Teaser

This quality improvement study examines outcomes of a multisite demonstration involving organizational strategies associated with registration and use of older adult patient portal shared access.

## Introduction

The patient portal allows patients to view sections of their electronic health record and perform health management tasks and is increasingly a mainstream modality for navigating care delivery demands, such as scheduling appointments and viewing test results.^1^ For patients who have difficulty accessing and using the patient portal (eg, those with low literacy, sensory or cognitive impairment, less technology experience), a care partner may assist with electronic health management activities.^2,3^ Many care delivery organizations allow patients to share access to their portal account by registering a care partner who then receives a unique login and password.^4^ Shared access respects patient information sharing preferences by allowing control over whom electronically interacts with clinicians on the patient’s behalf. At the same time, shared access supports care partners, providing them with access to information about patients’ health and a mechanism to navigate health system demands.^4,5^

A growing body of evidence has documented low uptake of shared portal access^6^ and that care partners often rely on patient credentials in portal interactions.^7,8,9^ Care partner use of the patient’s credentials to log into the portal may lead to data security and privacy problems, revealing more information than desired by the patient.^10^ Furthermore, this practice may contribute to confusion and mistakes if clinicians do not know with whom they are interacting electronically.^11^ As the patient portal may influence clinical decision-making and be used to complete legal documents, differentiating the identity of the person engaged in electronic health system interactions (the patient or someone else) is important in the delivery of safe and clinically appropriate care that is respectful of and responsive to patient preferences, as well as to learning health system initiatives that monitor care quality and outcomes in aging patient populations.^12,13^

We are unaware of prior organizational initiatives seeking to raise awareness and encourage registration for shared portal access.^14^ Thus, to address this gap, we undertook a multisite demonstration to test the outcomes of educational materials and implementation toolkits associated with registration for and use of shared access to the patient portal in routine care delivery. Drawing on date- and time-stamped portal interactions and surveys of patients and care partners, we evaluated the outcomes associated with the demonstration for care partner registration and use of shared access functionality.

## Methods

### Setting

In this single-arm, pre- and postobservation quality improvement study, the demonstration was conducted within diverse outpatient service delivery lines at 3 health care delivery organizations (sites) as previously described.^15^ Service delivery lines included a geriatric medicine service (site 1), a geriatric group spanning 3 primary care clinics (site 2), and a geriatric oncology consultation service (site 3). Each site received institutional review board approval for study activities. The electronic health record analysis involved a waiver of informed consent, and the survey included a prospective agreement in which respondents provided informed consent. The study adhered to the Standards for Quality Improvement Reporting Excellence (SQUIRE) reporting guideline.

The demonstration period spanned 12 months (July 1, 2022, through July 1, 2023, for sites 1 and 2), with a 6-month predemonstration period (January 1 through June 1, 2022) and 6-month postdemonstration period (August 1, 2023, through January 1, 2024). At site 3, the demonstration was delayed by 3 months. All 3 sites used Epic (Epic Systems) as their electronic health record vendor, with its accompanying MyChart patient portal. We developed a structured query language query to extract electronic health record data, which each site adapted to account for organizational differences in electronic health record data.

### Patient Population

Eligibility criteria included patients from partner sites who were (1) aged 65 years or older, (2) alive at the end of the demonstration, and (3) had 1 or more evaluation and treatment visits during the 12-month demonstration. Care partners who used the patient portal with shared access credentials were also included. We imposed evaluation and management visit inclusion criteria to ensure that the patients had an opportunity to receive the informational resources emphasized in the demonstration.

### Demonstration Rationale

The development of demonstration materials has been described in detail elsewhere.^15^ In brief, we used a human-centered design process to identify barriers to uptake, such as cumbersome registration processes, low awareness, and privacy concerns. Through stakeholder engagement, including patients, clinicians, and staff, we codesigned educational materials for patients, clinic staff, and clinicians, which were tailored to each organization’s branding and included multiple modalities (brochures, posters, quick response codes, etc) to improve accessibility. The demonstration also aligned with existing workflows by integrating shared access discussions into routine clinic interactions, such as appointment reminders, in-office visits, and after-visit summaries. By streamlining the registration process and providing accessible resources, the aim of the demonstration was to overcome barriers to care partner registration and support the broader use of patient portals, particularly for older adults.

### Study Design

We conducted a pre- and postobservation analysis of care partner registration and use of the patient portal by using electronic health record data. We also fielded a postdemonstration patient portal survey to patients and care partners to elicit information about shared access awareness and use.

### Measures

#### Patient Portal Registration and Use

Our primary end point was shared access registration data collected from time- and date-stamped electronic interactions. We compared the proportions of patients with a newly registered care partner for each of the 3 observation windows (6 months before demonstration launch, 12-month demonstration period, 6 months after the demonstration). We also assessed the proportion of patients newly registered for a patient portal. For the subset of eligible patients and care partners registered for the patient portal, we gathered data on use of portal functionality, including logins, laboratory results and clinical notes viewed, secure messages sent, and appointments scheduled, from time- and date-stamped electronic interactions for both patients and care partners for each of the 3 observation windows.

#### Awareness and Use of Shared Access

We assessed patient and care partner awareness and use of shared (proxy) access through patient portal surveys fielded by each site. The survey was hosted in REDCap and sent electronically to eligible patient portal accounts (including registered care partners) at each site (survey questions provided in the eAppendix in Supplement 1). At site 1, 2 rather than 1 visit were required before being sent the survey to adhere to organizational policies involving patient portal messaging for research.

### Patient Characteristics

Sex, age, and race and ethnicity were extracted from the electronic health record. Sex was categorized as female or male. Age was categorized as younger than 85 years or 85 years or older. Race and ethnicity were generally entered by clinic staff and not patient reported. In this study, race was categorized as White and other groups, including American Indian, Alaska Native, Asian, Black, Native Hawaiian or Pacific Islander, and missing. Ethnicity was categorized as Hispanic or non-Hispanic. Race and ethnicity were included to assess for differences in the demonstration’s uptake. Dementia diagnosis was also extracted from the electronic health record. Dementia was categorized using *International Statistical Classification of Diseases, Tenth Revision* codes defined by the Grodstein/Bynum method.^16^

### Statistical Analysis

We conducted descriptive analyses using R, version 4.4.1 (R Foundation). All differences were considered significant at a 2-sided *P* < .05, as we did not assume that portal registration and use for both patients and care partners would exclusively increase. We used χ^2^ tests to quantify the statistical significance of differences in the proportion of patients with a care partner who registered to access their patient portal account, comparing the 6 months before the demonstration period with the 6 months after. We used the Wilcoxon signed rank test, as data did not follow a normal distribution, to compare use of portal functionality (logins, secure messages sent, laboratory results and clinical notes viewed, and visits scheduled) through patient and shared access credentials (separately) in the 6 months preceding vs following implementation of the demonstration. We also compared new shared access registration among patients and care partners who had an existing registered patient portal account with those who registered for a patient portal account more recently (in the 6 months before the demonstration). Finally, we described patient- and care partner–reported awareness and use of shared access from survey responses using summary statistics. Missing data were not imputed.

## Results

### Patient Characteristics

A total of 16 005 patients from the 3 sites met the inclusion criteria with at least 1 evaluation and management visit during the 12-month demonstration period. Most patients (71.8%) originated from site 2, which included 3 participating primary care clinics, and most were younger than 85 years (84.8% vs 15.2% aged ≥85 years), female (61.5% vs 38.5% male), White (83.5% compared with 16.5% in the Other race category), non-Hispanic ethnicity (72.2% compared with 2.0% of Hispanic ethnicity), and enrolled in Medicare (92.5%) (Table 1).

**Table 1.** Characteristics of Participating Site Service Delivery Lines and Candidate Patients^a^

Variable	Sites or patients, No. (%)			
	Site 1, geriatrics	Site 2, primary care	Site 3, geriatric oncology	Total
**Service delivery line characteristics**				
No. of clinics	1	3	1	5
No. of clinicians at site	12	34	8	54
No. of clinic staff	5	93	15	113
Geographic location	Urban	Urban	Urban	NA
Academic (teaching) status	Major	Minor	Major	NA
Profit status	Nonprofit	Church-operated nonprofit	Nonprofit	NA
Organization EHR vendor type	Epic (2012)	Epic (2011)	Epic (2010)	NA
**Patient characteristics**				
No. of patients	3788	11 490	727	16 005
Age group				
<85 y	3028 (79.9)	10 068 (87.6)	483 (66.4)	13 579 (84.8)
≥85 y	760 (20.1)	1422 (12.4)	244 (33.6)	2426 (15.2)
Sex				
Female	2477 (65.4)	7016 (61.1)	348 (47.9)	9841 (61.5)
Male	1311 (34.6)	4474 (38.9)	379 (52.1)	6164 (38.5)
Race				
White	3311 (87.4)	9437 (82.1)	618 (85.0)	13 366 (83.5)
Other^b^	477 (12.6)	2053 (17.9)	109 (15.0)	2639 (16.5)
Ethnicity				
Hispanic	NA^c^	260 (2.3)	20 (2.8)	280 (2.0)
Non-Hispanic	NA^c^	10 847 (94.4)	707 (97.2)	11 554 (72.2)
Dementia diagnosis^d^	301 (8.0)	358 (3.1)	28 (3.8)	687 (4.3)
Medicare	3503 (92.5)	10 581 (92.1)	724 (99.6)	14 808 (92.5)
**Portal use (among registered users)**				
Activated patient account	3433 (90.6)	10 474 (91.2)	657 (90.4)	14 564 (91.0)
Activated shared access account	931 (24.6)	570 (5.0)	143 (19.7)	1644 (10.3)
Used patient portal account^e^	3124 (91.0)	9845 (94.0)	612 (88.0)	13 581 (93.1)
Used shared access patient portal account	859 (92.3)	151 (26.5)	103 (72.0)	1113 (67.7)

Abbreviations: EHR, electronic health record; NA, not applicable.

^a^ To be eligible, patients needed an evaluation and management visit during the 12-month demonstration period.

^b^ The other race category included American Indian, Alaska Native, Asian, Black, Native Hawaiian or Pacific Islander, and missing.

^c^ Ethnicity data were not available at site 1; thus, site 1 was not included in the denominator for this measure.

^d^ Dementia was defined using *International Statistical Classification of Diseases, Tenth Revision* codes drawn from the Grodstein/Bynum method.

^e^ Defined as an account that someone logged into at least once; the denominator is individuals who were registered for a patient portal account.

### Patient Portal Use

Most of the 16 005 patients had an activated patient portal account (91.0%) and logged into the account at least once (84.5%) during the 24 months spanning the demonstration period and pre- and postobservation periods (Table 1). Few patients had a registered care partner (1651 [10.3%]). Within this group, 1114 (67.5%) of registered care partners logged into their account. For the entirety of the 24-month observation period, patients sent a mean (SD) of 13.2 (26.1) secure messages, and care partners sent 0.3 (3.3) secure messages.

### Outcomes of the Demonstration Associated With Shared Access

**Figure 1.**
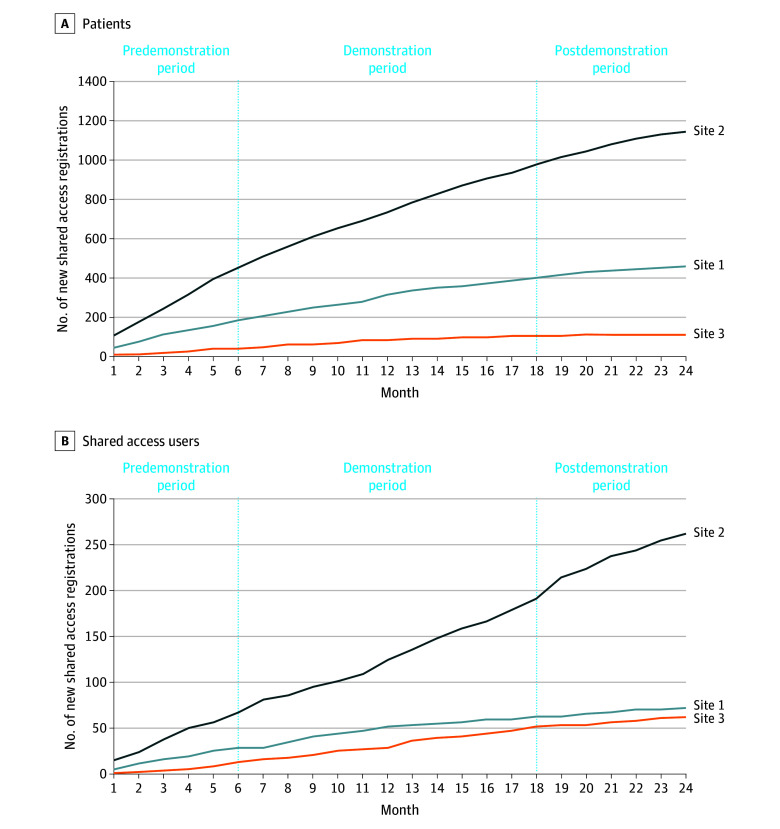
New Portal Registrations for Patients and Shared Access Users The slope was determined using the Microsoft Excel, version 16.88 (Microsoft Corp) slope function, which returns the slope of the linear regression line through data points in the known y-axis and known x-axis coordinates.

The percentage of patients with a newly registered care partner was comparable during the pre- and postdemonstration periods (0.7% [110 of 14 758] and 0.6% [91 of 14 016], respectively) (Table 2; Figure 1). The percentage of patients who newly registered for the patient portal decreased from 21.5% (677 of 3158) in the predemonstration period to 13.2% (225 of 1520) in the postdemonstration period (*P* < .001). The percentage of patients with a newly registered care partner was 2.6% (16 of 617) for those who registered for a patient portal account during the 6 months before the demonstration compared with 1.2% (139 of 11 680) of those who had an existing patient portal account.

**Table 2.** Patient Portal Use Before and During the Demonstration Period

Patient portal activity	Demonstration period, mean (SD)			Mean difference (95% CI)	*P* value
	6 mo Before	12 mo During	6 mo After		
**Patient activity**					
New registrations, No. (%)^a^	677 of 3158 (21.5)	814 of 2451 (32.9)	225 of 1520 (13.2)	χ^2^ = 26.1	<.001
Logins^b^	5.1 (8.4)	5.1 (8.1)	4.8 (8.2)	−0.14 (−0.28 to −0.02)	.03
Secure messages sent^b^	0.8 (1.8)	0.9 (1.8)	0.8 (1.5)	0.07 (0.05 to 0.10)	<.001
Laboratory results viewed^b^	1.5 (2.9)	1.5 (3.0)	1.4 (3.5)	0.05 (−0.01 to 0.11)	.74
Clinician notes viewed^b^	0.3 (1.2)	0.4 (1.4)	0.4 (1.7)	0.07 (0.05 to 0.10)	<.001
Visits scheduled^b^	0.9 (1.6)	0.9 (1.6)	0.8 (1.5)	0.01 (−0.02 to 0.04)	.64
**Shared access activity**					
New registrations, No. (%)^a^	110 of 14 758 (0.7)	195 of 14 594 (1.3)	91 of 14 016 (0.6)	χ^2^ = 1.08	.58
Logins^b^	5.9 (11.4)	6.2 (11.7)	6.8 (14.1)	1.34 (0.76 to 1.92)	<.001
Secure messages sent^b^	0.2 (0.9)	0.2 (0.6)	0.2 (0.8)	0.01 (−0.04 to 0.06)	.70
Laboratory results viewed	0.7 (2.7)	1.1 (3.4)	1.1 (3.7)	0.41 (0.21 to 0.61)	<.001
Clinician notes viewed^b^	0.2 (1.1)	0.7 (5.6)	0.6 (3.2)	0.36 (0.21 to 0.52)	<.001
Visits scheduled	0.8 (10.8)	1.2 (9.3)	1.0 (5.4)	0.22 (−0.31 to 0.76)	<.001

^a^ The overall sample included 16 005 patients from 3 health systems of whom 3158 and 1520 were not registered for the patient portal before the pre- and postdemonstration periods. A total of 14 758 and 14 016 patients did not have a registered care partner before the pre- and postdemonstration periods. These numbers are the denominators for the patient and care partner registrations, respectively.

^b^ Mean per month over the observation period among the subset of patients still alive and registered for the patient portal.

**Figure 2.**
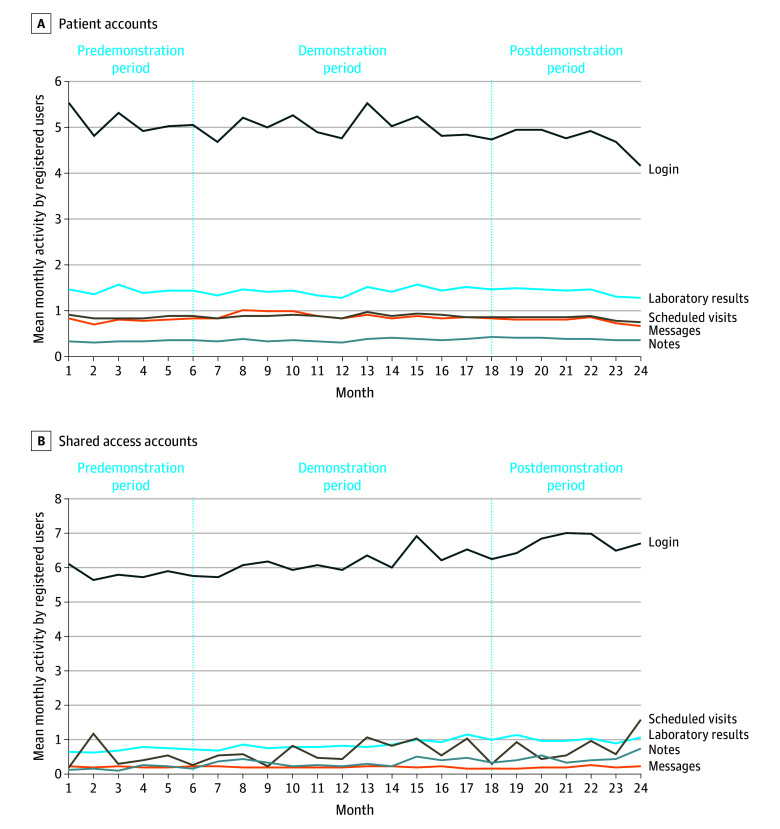
Trends in Patient Portal Use Across Patient and Shared Access Accounts

Care partner logins to the patient portal were higher during the post- vs predemonstration period (mean [SD], 6.8 [14.1] vs 5.9 [11.4]; *P* < .001) (Table 2; Figure 2). Similarly, care partners were more likely to view laboratory results (mean [SD], 1.1 [3.7] vs 0.7 [2.7]) and clinician notes (mean [SD], 0.6 [3.2] vs 0.2 [1.1]) and schedule visits (mean [SD], 1.0 [5.4] vs 0.8 [10.8]) in the post- vs predemonstration period (*P* < .001 for both contrasts). Patient portal activity did not differ across the 6-month pre- and postobservation periods for secure messaging, viewing laboratory results, or scheduling visits. However, more patients viewed clinician notes (mean [SD], 0.4 [1.7] vs 0.3 [1.2]; *P* < .001), and fewer patients logged in (mean [SD], 4.8 [8.2] vs 5.1 [8.4]; *P* = .04) during the post- vs predemonstration period.

### Patient- and Care Partner–Reported Awareness and Use of Shared Access Registration

At the end of the demonstration period, a total of 11 650 patients and care partners were invited to complete an electronic survey about awareness and use of the patient portal (eAppendix in Supplement 1), of whom 1755 (15.1%) responded (Table 3). Most respondents (1664 [94.8%]) identified as patients; few (91 [5.2%]) identified as care partners. A total of 721 patient respondents (43.3%) reported awareness of shared access. Of this group, 275 (38.1%) stated that they learned of shared access from site clinicians or staff or registered a care partner for their account (246 [34.1%]).

**Table 3.** Patient and Care Partner Report of Awareness and Use of Proxy Access Across 3 Health Systems

Reported awareness, use, and characteristics	Respondents, No. (%)			
	Site 1 (n = 410)	Site 2 (n = 1280)	Site 3 (n = 65)	Total (n = 1755)
Total messaged (response rate, %)	3060 (13.4)	8092 (15.8)	498 (13.0)	11 650 (15.1)
Patient respondents				
Total	361 (88.0)	1243 (97.1)	60 (92.3)	1664 (94.8)
Aware of shared access	204 (56.5)	483 (38.8)	34 (57.6)	721 (43.3)
Learned about shared access at site^a^	70 (34.3)	195 (40.4)	10 (29.4)	275 (38.1)
Registered care partner^a^	99 (48.5)	128 (26.5)	19 (55.9)	246 (34.1)
Shared access granted ≥1 y ago^b^	85 (85.8)	106 (82.8)	9 (60.0)	200 (82.6)
Relationship of shared access user to patient^b^				
Spouse	39 (39.4)	81 (63.2)	7 (46.7)	127 (52.5)
Adult child	48 (48.5)	38 (29.7)	5 (33.3)	91 (37.6)
Other^c^	11 (11.1)	9 (7.0)	3 (20.1)	22 (8.8)
Care partner respondents				
Total	49 (12.0)	37 (2.9)	5 (7.7)	91 (5.2)
Relationship to patient				
Spouse	22 (44.9)	11 (30.0)	1 (20.0)	34 (37.4)
Adult child	21 (42.8)	17 (46.0)	3 (60.0)	41 (45.0)
Other	6 (12.2)	9 (24.3)	1 (20.0)	16 (17.6)
Method of logging in				
Patient credentials	23 (46.9)	22 (59.4)	3 (60.0)	48 (52.7)
Shared access	18 (36.7)	12 (32.4)	1 (20.0)	31 (34.1)
Varies	8 (16.3)	3 (8.1)	1 (20.0)	12 (13.2)
Registered ≥1 y	49 (100.0)	34 (91.9)	5 (100.0)	88 (96.7)
Learned about shared access at site	25 (53.2)	17 (46.0)	1 (20.0)	43 (47.2)
Primary language spoken by care partner and patient				
English, patient does not	11 (22.4)	7 (18.9)	1 (20.0)	19 (20.9)
Both English	36 (73.5)	30 (81.1)	4 (80.0)	70 (76.9)
Patient and care partner respondents				
Remember receiving written materials about shared access at site	38 (9.3)	73 (5.7)	9 (14.1)	120 (6.8)
Remember speaking with staff about shared access at site	53 (12.9)	71 (5.0)	9 (14.1)	133 (7.6)

^a^ “Learned about shared access at site” and “registered care partner” were only asked if the patient responded yes to “aware of shared access.”

^b^ “Shared access granted ≥1 year ago” and “relationship of shared access user to patient” were only asked if the patient responded yes to “had someone sign up for shared access.”

^c^ Other care partner relationships with patients included close friends and neighbors.

Most of the 91 care partners identified as spouses (34 [37.4%]) or adult children (41 [45.0%]) (Table 3). Nineteen care partners (20.9%) reported that they assist a patient who does not speak English. Forty-eight care partners (52.7%) reported using patient credentials to access the portal, and 31 (34.1%) reported using their own identifying credentials, with the remainder (12 [13.2%]) reporting that it varies. Forty-three care partners (47.2%) reported learning about the patient portal from site clinicians or staff.

## Discussion

This multisite quality improvement study of a novel initiative assessed the outcomes associated with organizational strategies to overcome identified barriers to shared portal access awareness and registration of care partners. We found that the initiative did not increase new shared access registration to the patient portal and modestly increased care partner use of portal functionality. The trend in new shared access registrations to the patient portal was stable throughout and after the demonstration period, whereas new patient registrations decreased. However, more than one-half of patients reported not being aware of shared access at the end of the demonstration period.

Our initiative was the product of extensive community engagement in developing outreach strategies, communication and branding materials, and site-specific workflows that might resonate with diverse users, and it was undertaken in partnership with committed and enthusiastic sites.^15^ Results are thus sobering and suggest the limits of public awareness and clinician and staff education alone in overcoming technical, informational, and workflow challenges of engaging care partners through the patient portal.^17,18^ For example, signing up for shared access can be cumbersome if the care partner is not also a patient at the same health system, which may deter care partners,^17^ and patient portal sign-up for care partners may be difficult to prioritize at a busy primary care clinic.

This multisite study is the first of shared portal access functionality, and we observed notable variation in rates of registration and use across sites. The higher uptake of shared access within geriatric and oncology service delivery lines (vs general primary care), aligns with conclusions from a recent scoping review that patient capacity and health are key factors in motivating care partner portal use.^6^ Differences in organizational policies were observed to have ramifications for shared portal registration. The site with the highest registration followed a policy of responding to nonpatient authors of patient portal messages (involving patient credentials) with a warning of account deactivation if improper use of credentials in messaging continued. Our findings collectively reinforce the role of organizational policies in digital health engagement and suggest the potential value of organizational learning collaboratives and policy guidance to support best practice implementation strategies in this arena.

Clinicians and staff have experienced increasing workloads associated with messaging in recent years, raising concerns about career satisfaction and burnout.^19,20^ Busy clinicians wary of any additional time demands resulting from engaging with care partners should be reassured that although registered care partners commonly accessed the patient portal to view patient health information, few engaged in direct messaging. Though our study was not able to quantify outcomes associated with the demonstrations for clinicians, clinicians from participating sites reported benefits from the ability to distinguish with whom they are messaging (a patient or their care partner) as potentially reducing time demands due to care partners being able to directly access patient health information. Informational videos sent directly to patients’ portal accounts and webpages with information on shared access that are easy to access from health systems’ websites may be ways to reach care partners without burdening clinical staff.^21^

Contrary to common assumptions about digital literacy and access among older adults, we observed high patient portal use in this patient population. Older adults commonly desire or rely on care partner involvement when navigating care delivery demands.^22^ As many patients who rely on a care partner may be vulnerable, supporting care partners’ use of the patient portal may be a way to bridge gaps in access.^6,23^ Care partner engagement is a policy and practice priority in achieving quality in age-friendly care,^24^ the hospital discharge process,^25,26^ and the care of persons living with dementia.^27,28^ Care partners are notoriously undersupported by health care systems,^29,30^ and shared access merits policy attention as an existing functionality that offers them greater legitimacy and information transparency in health system interactions while being respectful to the privacy preferences of the patient.^31^ Legal documents and other sensitive health information are exchanged over the patient portal, and without consistent use of shared access, patient privacy and wishes may be violated.^4,5^ It is also notable that patient and care partner responses to patient-facing questionnaires differ and cannot be conflated^32,33^ given the role of the patient portal in clinical decision-making.

Changes at the policy level are needed to drive shared access use. Organizations including the Joint Commission on Accreditation of Healthcare Organizations and the Office of the National Coordinator for Health Information Technology are well positioned to advocate for integration of shared access implementation in reporting and credentialing activities. Federal and state governmental agencies tasked with monitoring the spread of consumer-facing health information technologies might evaluate both patient and family adoption when tracking diffusion and use. Accreditation organizations might incorporate metrics of shared access availability and uptake in evaluating quality for particular patient subgroups (eg, patients with dementia) or programs (eg, patient-centered medical homes) related to consumer engagement. Until shared access is appropriately recognized as a resource that organizations should be actively supporting and enforcing, it may continue to be an underused resource.

### Limitations

Our study is subject to design limitations. As the evaluation did not include a comparison group, interpretation of results may be subject to biases associated with differential portal registration and use by key subgroups.^2,34,35^ The lack of a comparison group inhibited our ability to assess what differences in use and registration were attributable to time. As the 3 sites had predominantly White, non-Hispanic patient populations, we were unable to examine differences by race and ethnicity in shared access uptake. We lacked information about how many patients did not have a care partner who would have been an appropriate shared access user or, vice versa, how many patients’ care partners were using patient credentials for whom the demonstration could not reach.

Key outcomes were ascertained from electronic health record data, which are subject to measurement error and misclassification.^36^ Death is not consistently captured in electronic health records and typically not missing at random.^37^ We sought to maximize comparability in operational definitions and measurement of patient portal interactions for our 3 partner sites, but unobserved site differences may have been present nonetheless. Our partner sites were selected for their strong commitment to patient and family engagement and sophisticated informatics teams, and results may not generalize to mainstream care. The response rate to the survey was low (15%), with few care partner respondents, and responses were not identifiable, which limits our ability to generalize survey findings.

## Conclusions

This multisite quality improvement study found modest success in increasing patient portal shared access registration using educational strategies. Our demonstration was largely focused on individual service delivery lines as touchpoints where the patients and care partners could learn and register for shared access. Shared access registration remained consistent throughout the demonstration period and afterward, while general patient portal registration decreased.

## Notes

Supplement 1. **eAppendix.** Survey Questions

Supplement 2.
Nonauthor Collaborators

Supplement 3.
Data Sharing Statement
