# Supplementary material for: A Multisite Demonstration of Shared Access to Older Adults’ Patient Portals
Source: JAMA Netw Open. 2025 Feb 25;8(2):e2461803. doi: 10.1001/jamanetworkopen.2024.61803 (PMC11862967; doi:10.1001/jamanetworkopen.2024.61803)
Supplement: Supplement 1. — eAppendix. Survey Questions [file jamanetwopen-e2461803-s001.pdf]

Supplemental Online Content

Gleason KT, DesRoches CM, Wu MMJ, et al. Evaluation of a multisite demonstration to increase shared access to older adults' patient portals. *JAMA Netw Open*. 2025;8(2):e2461803. doi:10.1001/jamanetworkopen.2024.61803

eAppendix. Survey Questions

This supplemental material has been provided by the authors to give readers additional information about their work.

© 2025 Gleason KT et al. *JAMA Network Open*.

eAppendix. Survey Questions

Please complete the survey below. Thank you!

Are you	◯ The patient registered to this MyChart account<br>◯ Someone other than the patient
What is your relationship to the patient?	◯ Spouse<br>◯ Adult Child<br>◯ Other Family<br>◯ Close Friend<br>◯ Other: ______
How many times did you access your MyChart in the last 12 months?	◯ Never<br>◯ 1-2 times<br>◯ 3-5 times<br>◯ 6-9 times<br>◯ 10+ times
How many times did you access the patient's MyChart in the last 12 months?	◯ Never<br>◯ 1-2 times<br>◯ 3-5 times<br>◯ 6-9 times<br>◯ 10+ times
How do you usually log in to the patient's MyChart?	◯ I usually log in as the patient, with the patient's login/password<br>◯ I usually log in as myself<br>◯ It varies
How long have you had access to the patient's MyChart account?	◯ Less than one last year<br>◯ Longer than 1 year, but less than 5 years<br>◯ 5 or more years
How did you learn about MyChart?	◯ I heard about it from the clinicians and staff at the XX clinic<br>◯ I heard about it from clinicians and staff at a different clinic<br>◯ Other
Please describe how you learned about MyChart:	__________________________________
The University of XX MyChart patient portal offers patients the option of giving another person, such as a spouse, adult child, other family, or friend, access to their medical record through MyChart. This is called Proxy Access. With Proxy Access, someone other than the patient has their own login credentials (user name and password) separate from the patient.<br><br>If you signed up for Proxy Access, please tell us about your experience getting signed up and using MyChart.<br><br>If you did not sign up, why not?	____

© 2025 Gleason KT et al. *JAMA Network Open*.

The University of XX MyChart patient portal offers patients the option of giving another person, such as a spouse, adult child, other family, or friend, access to their medical record through MyChart. This is called Proxy Access. With Proxy Access, someone other than the patient has their own login credentials (user name and password) separate from the patient.<br><br>Before today, were you aware that University of Utah offers Proxy Access?	◯ Yes<br>◯ No
Have you ever allowed a family member or friend to sign up for Proxy Access to your MyChart account?	◯ Yes<br>◯ No
What is their relationship to you?	◯ Spouse<br>◯ Adult Child<br>◯ Other Family<br>◯ Close Friend<br>◯ Other
If you selected "other", please describe their relationship to you:	__________________________________
When did you grant your [their_relationship] access to your MyChart account?	◯ Less than one last year<br>◯ Longer than 1 year, but less than 5 years<br>◯ 5 or more years
Where did you learn about MyChart Proxy Access?	◯ I heard about it from the clinicians and staff at the XX clinic<br>◯ I heard about it from clinicians and staff at a different clinic<br>◯ Other
If you selected "other", where did you learn about MyChart Proxy Access?	__________________________________
If you had a care partner sign up for Proxy Access, please tell us about your experience with getting the care partner signed up and using MyChart. If your care partner did not sign up, why not?	____
In the last year at XX Clinic, do you remember receiving written materials about MyChart Proxy Access?	◯ Yes<br>◯ No
In the last year at XX clinic, do you remember speaking to the clinicians or staff about MyChart Proxy Access?	◯ Yes<br>◯ No
In the last year at XX Clinic, please describe any experience speaking with clinic staff about MyChart Proxy Access or receiving any written materials about it.	____
What is your age in years?	__________________________________

© 2025 Gleason KT et al. *JAMA Network Open*.

In general, how would you rate your overall health?	◯ Excellent<br>◯ Very good<br>◯ Good<br>◯ Fair<br>◯ Poor
What is the highest grade or level of school that you have completed?	◯ Did not graduate from high school<br>◯ High school graduate or GED<br>◯ Some college credit, but no degree<br>◯ Associate's degree<br>◯ 4-year college degree<br>◯ Master's, professional or doctorate degree
How confident are you about your ability to take good care of your health?	◯ Completely confident<br>◯ Very confident<br>◯ Somewhat confident<br>◯ A little confident<br>◯ Not confident at all
What are the primary languages spoken by you and the patient?	◯ My primary language is English, the patient's primary language is other than English<br>◯ My primary language is English, the patient's primary language is also English<br>◯ My primary language is not English, the patient's primary language is English<br>◯ My primary language is not English, the patient's primary language is also not English
What is your primary language?	◯ English<br>◯ Spanish<br>◯ Other language

© 2025 Gleason KT et al. *JAMA Network Open*.
