# Supplementary material for: A Multisite Demonstration of Shared Access to Older Adults’ Patient Portals
Source: JAMA Netw Open. 2025 Feb 25;8(2):e2461803. doi: 10.1001/jamanetworkopen.2024.61803 (PMC11862967; doi:10.1001/jamanetworkopen.2024.61803)
Supplement: Supplement 2. — Nonauthor Collaborators [file jamanetwopen-e2461803-s002.pdf]

Supplemental Online Content: Nonauthor Collaborators
*First name, last name, and suffix (if applicable) are required and will appear in PubMed.

*Group Name(s): Shared Access Project Team							
***First Name and Middle Initial(s)**	***Last Name**	***Suffix (eg, Jr, III)**	Academic Degrees	Institution	Location (city, state/province, country)	Role or Contribution, eg, chair, principal investigator	Group (if more than 1 Group listed in the byline) and/or Subgroup (eg, Steering Committee)
Elizabeth	Salmi			OpenNotes			
Martha	Kenyon			University of Rochester			
Deborah	Wachenheim			OpenNotes			
Kathleen	Fear			University of Rochester			
