# Supplementary material for: A Multisite Demonstration of Shared Access to Older Adults’ Patient Portals
Source: JAMA Netw Open. 2025 Feb 25;8(2):e2461803. doi: 10.1001/jamanetworkopen.2024.61803 (PMC11862967; doi:10.1001/jamanetworkopen.2024.61803)
Supplement: Supplement 3. — Data Sharing Statement [file jamanetwopen-e2461803-s003.pdf]

Data Sharing Statement

Data
Data available: Yes
Data types: Deidentified participant data
How to access data: Data may be requested through an email to the corresponding author.
When available: With publication

Supporting Documents
Document types: None

Additional Information
Who can access the data: Researchers whose proposed use of the data has been approved.
Types of analyses: For a purpose related to understanding portal use among older adult patients.
Mechanisms of data availability: After approval of the proposal.
